# Dental Embryology

**Published:** 1903-01-15

**Authors:** G. S. Blanchard


### DENTAL REGISTER.

Vol. LVII. January 15, 1903. No. 1.

### DENTAL EMBRYOLOGY.

BY G. S. BLANCHARD, D.D.S.

Read before the Ohio State Dental Society, December 3, 1902.

The subject of dental embryology does not seem to have much bearing upon the every-day practice of dentistry, and possibly does not make much difference in plate-work, or in crown and bridge-work, or for that matter, in any work on the tissues of the adult. The adult portion of our practice is the larger portion, the most remunerative portion, and the most satisfactory portion in every way. Once in a while a dentist is found who does not mind working for children, and even says that he likes it, but he is a "rara avis," and we are inclined to think he is prevaricating when he makes the statement. Most of us do what is necessary for children and are glad it is no more and sometimes are inclined to stretch our consciences in the matter of what is necessary.

But nevertheless, the child's teeth should receive even more studious consideration than those of the adult, because (applying the old saying "As the twig is bent so the tree is inclined,") upon the child's teeth and the care they receive, depends in a large measure the condition of the mouth in after years. In what way is this true? First and foremost, in the matter of the extraction of temporary teeth, premature extraction resulting in the destruction of the permanent tooth germ or more frequently in irregularities such as mal-position, impaction, etc., with their accompanying disorders and discomforts, also in the treatment of decayed temporary teeth where the pulp is exposed. Knowledge of the extent of calcification at the apex is very valuable. Lastly in the care of the first, second and third permanent molars.

These, to my mind, constitute the practical side of the subject, (the ability to decide what to do, depending upon the knowledge of the situation). A few rules and tables of when teeth erupt will cover the ground. But the kind of knowledge gained from such a source is blind. The kind which grasps the situation understandingly is far more satisfactory and insures better results. But there is another reason why we should be interested in this subject. The salvation and preservation of the human tooth, is the focal point upon which all our energies are concentrated, and it seems to me that anything pertaining to the origin and growth of teeth should command our immediate attention, and knowledge on this subject be eagerly sought after. How can this kind of knowledge be obtained? In no other way than by studying the conditions as they exist previous to eruption. I have thought and worked some along this line for some time past, and when I decided to appear before you I expected to use some of my own illustrative material. Upon looking more carefully into the subject I decided that with what material I had in shape, I could not expect to hold your interest.

I shall treat of the subject macroscopically, citing only a few microscopic specimens to show early conditions, edeavoring to give an idea of the various forms of the organs in the process of their development.

The first preparations for the development of the teeth take place about the middle of the second foetal month, when following the line of the future alveolar ridge a slight heaping up of the epithelium is noticed. Immediately beneath this is a dipping in of the deep epithelial layers in the direction of the future alveolus. These constitute what is known as the tooth band. As it passes more deeply into the tissue it assumes the form of a letter V with the open end toward the surface. Shortly after this there appears on the inner or concave surface of the band, a thin membranous plate extending the entire length of the band. This plate or lamina springs from the band at a point midway between its border and base. From this lamina are given off ten little buds which develop into the germs for the temporary teeth. These buds lengthen into cords which spread laterally at the ends.

We find that the enamel organ arises from the oral epithelium while the dentin is formed from the connective tissue of the jaw. As the enamel organ recedes from the surface, the concavity increases and the bell-shaped outline becomes more clearly defined. Four layers of cells constitute the enamel organ. First, external epithelium; second, stellate reticulum (occupying the center of this space); third, stratum intermedium; fourth, internal epithelium or ameloblastic layer. From this last the enamel is calcified. Enamel calcification is the chief function of the enamel organ, but many writers think its primary function is to mold the tooth-form. This however does not seem to be true. The enamel organ does not assume the shape of the future tooth, but in most instances the dentin organ does assume that shape. The dentin germ is composed of elements which result in a highly vascular, compact tissue. The enamel organ on the contrary is a gelatenous-like mass which would readily succumb to pressure from the active connective tissue cells within. The dentin organ originates from the connective tissue of the primitive jaw and as it pushes up into the enamel organ and becomes enveloped by it, assuming the shape of the future tooth crown, there is a rapid change in its structure. It becomes more vascular—its peripheral cells differentiate and the essential dentin forming cells, the odontoblasts appear. These are in close relation to the ameloblasts or enamel forming cells and the two together form the future tooth crown. The papilla or dentin organ decreases in size as calcification progresses and eventually becomes the tooth pulp. Thus calcification proceeds in two directions simultaneously, beginning at the line of division of enamel and dentin and proceeding toward the surface and at the same time towards the interior. The dentin forming cells or odontoblasts lay down their initial layers first, closely followed by the ameloblasts.

The lamina from which spring the germs for the temporary teeth is given off from the tooth-band at a point between the base and the upper border, leaving a free margin. From this margin is generated the enamel organs of the succedaneous teeth. But this does not account for the permanent molars, which are not succedaneous. Some think that as the jaw lengthens, the band extends backwards giving off buds successively for the first, second and third molars. Another theory is that they are derived directly from the sub-epithelial tissue. However this may be, the subsequent growth is identical to that of the temporary teeth.

As the development of the tooth germ proceeds it becomes definitely separated from the surrounding tissue by what is known as the "dental follicle," sometimes called tooth-sac or sacculus. This membrane is developed by a proliferation of cells at the base of the dental papilla and extends first over the papilla and then over the enamel organ, becoming firmly attached to the deep layer of the surface epithelium. Thus the tooth germ is found swinging in a membraneous pocket attached to the surface epithelium by a band only. The follicular wall is composed of two layers, the outer one is dense and firm and becomes the dental periosteum. The inner one is thin and frail and later on assists in the formation of the cementum. About the fourth fetal month the band is broken and the second and saccular stage of tooth development is reached. Centers of ossification for the maxilla appear at an early date, about the sixth week for the mandible and the eighth for the upper jaw.

A section of the lingual aspect of the lower jaw of a three months fetus will show the remains of Meckel's cartilage much wasted. This cartilageous band appears in the mandible at the beginning of the second month and forms a framework around which ossification takes place. Later a somewhat similar cartilage appears in the upper jaw. The cartilage itself does not become calcified but disappears by the seventh month, except the portion near the tympanum, which is ossified into the malleus.

A section at birth will show a gradual increase in length and but little change in contour. The length is increased by osseous deposits at the distal extremity.

Outwardly the bone changes but little, but within there is a complete transformation: first, a simple gutter is seen in which the follicles hang, but at birth each follicle is enclosed in its individual crypt except the second molar, which is not divided from the first permanent molar 'till later. As the follicles increase in size they become more perfectly enclosed, the bone almost closing over the teeth. No sooner have they reached this stage than resorptive action takes place as the crown advances and they disappear, appearing again at the evolution of the permanent teeth; to be again swept away when they erupt.

The first visible signs of preparation for teeth, aside from those shown by dissection, are observed about the beginning of the third month, when a well-defined infolding of the oral epithelium may be seen. The roof of the mouth is well defined, the palate bones having united. The primitive dental groove marks the position of the tooth band.

We have now reached a period when external conditions have some bearing on the condition of the teeth.

Previous to this time the development has been going on, uninterrupted by any morbid conditions existant in the parent. Few irregularities occur up to this time. A short time before birth the condition of the permanent teeth is far enough advanced to permit of study.

There were different theories as to the origin of the succedaneous teeth. Some thought the buds for their germs came from the sacs of the temporary teeth, others that they were from the primitive cord immediately after its rupture at the time of the beginning of the saccular stage. It is now thought that the bud is given off from the primitive cord at a point in close proximity to its attachment to the deciduous enamel organ.

The permanent molars, come as nearly as we can find out, from the extension of the original tooth-band. Some think that the bud for the first permanent molar is given off from the second temporary molar, the second molar from the first and the third from the second.

As the temporary teeth advance the permanent one recede and are soon enclosed in separate crypts, only connected with outside tissue by the leading cord of the gubernaculum.

The papilla of the permanent tooth-sacs have already assumed the tubercular outline of the future cutting edge. There seems to be a firm connection between the temporary and permanent tooth-sacs. This is broken down when the permanent teeth bocome enclosed in their separate crypts.

At three months the sacs for the permanent incisors are almost as large as those of the temporary ones. Those of the cuspids and bicuspids are smaller owing to their later development. At this time the calcification of the crowns is about complete, externally and preparations for the roots have begun. The interior of the crown is not completely calcified however, this being a gradual, continuous process. The *crown* was molded upon the dentin papilla. So it is with the root. The sac gradually lengthens and the pulp grows correspondingly and is molded to the root form, calcification taking place by the generation of odontoblastic cells on the periphery. With two and three rooted teeth the laying down of the odontoblastic cells follows the bifurcation and trifurcation of the tooth pulp. The cementum or peri-cementum which surrounds the root is deposited by the inner layer of cells of the follicle. These are very similar to osteoblasts and are called cementoblasts. We noticed a while ago that the bone gradually grew about the follicle, first forming an open gutter, next surrounding on all sides and finally enclosing the tooth in a bony crypt. This condition is reached about the seventh or eighth month after birth. As soon as encasement is complete resorption begins, starting with the last-formed portion. This may be attributed to the advancement of the teeth, but in reality it is not generally understood. Ordinarily we suppose that eruption is caused by the addition of material to the free end of the tooth, but this is not true in the case of the cuspid, which is almost fully formed before it advances to the surface. In the process of eruption the labial surface is removed chiefly, the palatal or lingual surface remaining almost intact, allowing the tooth to glide past and out into position. This remaining portion also protects the forming permanent tooth beneath. As the tooth passes into its new position the process is built up again around it so that by the time it is fully erupted it is firmly supported by the newly formed bone. At the same time the roots are lengthening and are completed when the tooth is fully erupted or soon after.

Dr. C. N. Pierce has devised a chart showing calcification of both the temporary and permanent teeth, which is especially useful because by it we can tell the conditions obtaining at the apex of a temporary tooth which has an exposed pulp. No office should be without this chart.

The upper jaw of a two-year old child will show all the teeth erupted except the second molar. A dissection will show the second deciduous molar, first permanent molar and the permanent incisors in their sacs. The crowns will be observed to be heading in various directions, sometimes indeed, are found to be at right angles to their proper alignment. The gubernaculum here comes into play directing them into their proper places by the tension of its fibers and also the foramen which it creates in the bone over the teeth stimulating resorptive action in that direction.

By the close of the second year the twenty deciduous teeth are in position, roots fully calcified and apical foramina fully established. This exists for but a short while. About the fourth year decalcification begins again at the apices. It progresses in the same order that calcification took place, that is, in the order of their eruption. About three years elapse till nothing is left but the crowns and they are then shed. About the sixth year, the jaw having extended sufficiently, the first permanent molars have erupted.

Between the tenth and eleventh years the temporary molars are lost and the bicuspids appear. In another year, by further extension, the second permanent molar has room to erupt. Then follows the cuspid, making fourteen teeth in each jaw. The third molar has to await accommodations for itself in the lengthening of the jaw, usually erupting between the eighteenth and twenty-first years. It is sometimes delayed for a long time and occasionally is absent entirely.

We have passed hastily over the subject only glancing at the various phases and conditions of tooth formation, but in a general way have outlined a subject, which to me, is one of absorbing interest.

The field covered by the term "Dental Embryology" is a vast one. I have treated the subject macroscopically only and have left untouched many interesting things. When we consider the microscopic side of the question, we are in a wilderness where such eminent investigators as Williams and Sudduth confess themselves lost.

Much therefore remains to be done. Many laurels may be won in this most interesting field. Let us then address ourselves to the work. I am sure that our efforts will be rewarded in the satisfaction of knowing and seeing the wonderful processes through which the dental organs must pass before they take their positions in the mouth to do their proper work in the maintenance of health and strength of mankind.

